# Supplementary material for: Fiscal decentralization, local government innovation preference, and enterprise technological innovation: Evidence from China
Source: PLoS One. 2026 Apr 15;21(4):e0347025. doi: 10.1371/journal.pone.0347025 (PMC13082718; doi:10.1371/journal.pone.0347025)
Supplement: Attachment — Submitted filename: Responses to Reviewers.docx [file pone.0347025.s003.docx]

**Detailed Responses to Editor and Reviewers**

**Dear Editor and Reviewers,**

We are glad and grateful for the opportunity to improve our manuscript’s quality. We much appreciate your constructive comments, intellectually stimulating suggestions, and encouraging words. We have carefully considered all the comments and revised our manuscript accordingly and the changes and revisions made in this manuscript are marked in RED. Thank you for providing valuable and insightful comments, which indeed helped us to learn how to write a top-tier academic paper and how to enhance our manuscript. Thank you so much.

| **Valuable comments from Reviewer #1:** | |
| --- | --- |
| **Valuable comment 1**  The reviewed article examines an intriguing topic: the role of local governments in supporting innovation, particularly in enterprises they own. However, this issue is specific to China for two main reasons. First, local governments own state-owned companies (SOCs) that operate in private, and even international, markets—not just in areas related to municipal services. Second, the Chinese model of decentralization is distinct; it constitutes a form of delegation rather than true decentralization as defined by fiscal decentralization theory. While the authors touch on this in the article, it should have been emphasized in the title. Additionally, the article should explain why this topic might interest readers from other countries where such solutions do not exist. | **Response to valuable comment 1**  We thank the reviewer for highlighting the China-specific nature of our research context. We fully agree that the institutional setting examined in this paper is distinct from classical models of fiscal decentralization.  In response, we have revised the title (Please see P.2) and introduction (Please see P.2-3) to explicitly emphasize that China’s system represents a form of fiscal delegation under political centralization, rather than genuine fiscal decentralization in the federalist sense. We revise the title of the paper to “**Fiscal Decentralization, Local Government Innovation Preference, and Enterprise Technological Innovation: Evidence from China**”.  To this end, we have substantially revised the introduction section to explicitly emphasize the institutional distinctiveness of China’s decentralization framework. This revision highlights that China’s fiscal system is better conceptualized as a form of fiscal delegation under centralized political control, rather than a transfer of sovereign fiscal authority.  To further situate this study within an international comparative context, we have added relevant discussions that contrast China’s decentralization trajectory with those of other countries. These analyses illuminate why China’s provincial, municipal, and county-level fiscal systems serve as an exceptionally informative setting for examining how fiscal decentralization shapes local government behaviors and innovation incentives. |
| **The following contents in red are added, supplemented, or revised in the context of this paper**  **1 \| Introduction**  Under the background of diminishing marginal effect of monetary policy, the importance of finance for economic development and scientific and technological innovation has become the consensus of governments. As a national fundamental institutional arrangement of fiscal power distribution relationship (Wang et al. 2025), the in fiscal decentralization China implies a three-layer principal-agent relationship among the central government, local governments and enterprises (Tirole, 1986; Hu et al. 2024), which constitutes a de facto institutional constraint on the technological innovations of the enterprises through the government’s “tangible hand”. Second, due to differences in political and economic environments, fiscal decentralization practices vary across countries. In China, fiscal decentralization has played a particularly important role (Chen et al., 2026). The coexistence of political centralization and economic decentralization ensures central government control while aligning local government incentives with market economic development (Zhang, 2006; Jin et al., 2005). Decentralization reforms require sufficient institutional and state capacity (Chen et al., 2026). In Russia, the central government has gradually lost leadership during decentralization and failed to provide sufficient incentives for local development (Blanchard and Shleifer, 2001; Zhuravskaya, 2000). In many developing countries, decentralization is associated with insufficient public resource supply and increased inequality (Bardhan and Mookherjee, 2006). Against this backdrop, examining China’s local and county fiscal system provides practical experience for other centrally planned countries facing decentralization challenges.  …… | |
| **Valuable comment 2**  One issue is that the authors claim to focus on technology companies, but this is not evident in the sample presented. They refer to patents as a measure of technological innovation; however, patents can address a wide range of issues beyond just innovative technological solutions. It might be more appropriate to discuss innovative companies in general, rather than strictly focusing on innovations in new technologies. Alternatively, it could be helpful to clarify that this issue pertains specifically to new technologies. | We appreciate the reviewer’s insightful comment regarding the distinction between technology companies and the use of patents as a measure of technological innovation.  Following this suggestion, we have revised the manuscript (Please see P.8) to clarify that our analysis focuses on technological innovation activities at the firm level, rather than limiting the sample to firms classified as technology companies.  In addition, we have expanded the discussion in the variable definition section to explicitly acknowledge that patents may cover a broad range of innovative activities. We now emphasize that our study concentrates on patents related to new technologies, which are commonly used in the literature as a proxy for firms’ technology-oriented innovation outcomes. |
| **The following contents in red are added, supplemented, or revised in the context of this paper**  **3 \| Research Design**  **3.1 \| Variable Selection and Measurement**  **3.1.1 \| Explained Variables**  ……  Patent is an important indicator reflecting the technological innovation output of enterprises, which is characterized by easy accessibility, comparability and large amount of information. This paper draws on the practice of Wu and Wu (2025) and adopts the number of patent applications to measure the technological innovation of enterprises. This paper defines technological innovation as the innovative outcomes achieved through the development and application of new technologies. Although patents may cover various fields, this study specifically focuses on patents related to new technologies, which directly contribute to a company's technological advancement and market competitiveness. | |
| **Valuable comment 3**  Another concern is the lack of a reference that highlights a characteristic feature of the Chinese model of power division—namely, the competition among local authorities for the competition among local authorities for "points" from the central government. (see for example Yu, C., Hou, L., Lyu, Y., & Zhang, Q. (2022). Political competition, spatial interactions, and default risk of local government debts in China. Papers in Regional Science, 101(3), 717–743. Retrieved from https://doi.org/10.1111/pirs.12668) I believe this is a crucial factor in understanding why local authorities support actions favored by the central government, as evidenced by the quote at the line 522. | Thank you for this insightful comment. We agree that competition among local governments for performance “points” assigned by the central government is a distinctive and crucial institutional feature of the Chinese model of power division.  Following your suggestion, we have incorporated Yu et al. (2022) into the theoretical analysis section to explicitly characterize the horizontal competition among local authorities under a vertically centralized evaluation system. Specifically, we clarify that local governments not only respond to central government policy signals but also strategically adjust their behavior in comparison with peer jurisdictions in order to enhance their relative standing in the performance appraisal process.  This addition strengthens the institutional foundation of our argument regarding why local governments actively support actions favored by the central government, including innovation-oriented policies (see revised Section 2.2.1).Meanwhile, we have also made corresponding revisions in the relevant sections of the paper (Please see P.3 & P.18) . We appreciate this suggestion, which helps improve the theoretical completeness and contextual accuracy of the paper. |
| **The following contents in red are added, supplemented, or revised in the context of this paper**  **1 \| Introduction**  ……  This paper analyzes the existing literature from the technical innovation effect of fiscal decentralization and the influence factors of enterprise technological innovation in two aspects: First, the technical innovation effect of fiscal decentralization. The effect of fiscal decentralization on technological innovation is mainly analyzed by provincial and municipal local governments (Wang et al. 2022), and there are two distinct views: “decentralization inhibition theory” and “decentralization promotion theory” (Zallé and Bakouan, 2024). Scholars who support the “decentralization promotion theory” believe that fiscal decentralization is conducive to strengthening the government’s innovation function (Pu et al. 2023), and promoting the development of technological innovation activities of local governments and their efficiency (Zhao and He, 2024). Scholars who support the “decentralization inhibition theory” believe that fiscal decentralization will exacerbate the vicious competition between governments and make the investment structure of local governments show the obvious self-interested characteristic of “light on innovation and heavy on production” (Song et al. 2025; Zallé and Bakouan, 2024; Li et al. 2020). The reason why fiscal decentralization reduces the local government’s innovation preference is that, on the one hand, political competition among Chinese local officials is structured as a performance-based tournament overseen by higher-level authorities. Yu et al. (2022) provide systematic evidence that such competition leads to spatial interactions among politically connected jurisdictions, as local officials strategically adjust their behavior in response to their peers in order to improve their relative standing in the promotion hierarchy (Fang et al., 2025). Compared with the productive financial expenditures such as infrastructure, the innovative expenditures have the attribute of public goods, and this kind of behavior of “doing others a favor” is not in line with the interests of the local government. On the other hand, scientific and technological innovation is highly uncertain. Under the promotion championship mechanism of political centralization and economic decentralization, local government officials with the attribute of “politicians” tend to squeeze out the expenditures on unproductive public goods such as innovation in pursuit of economic growth during their tenure (Song et al. 2025).  ……  **2 \| Theoretical Analysis and Research Assumptions**  From the perspective of the evolution path of China’s fiscal system, before the 1994 tax-sharing reform, the fiscal system mainly acts directly on microeconomic subjects through embedding enterprises (especially state-owned enterprises). The tax-sharing reform has embedded China's finances in the economic cycle, forming a three-tier principal-agent relationship among the central government, local governments and enterprises. Local governments play a pivotal role in connecting the central government and enterprises in their jurisdictions. While the three-tier principal–agent framework provides a useful analytical lens, its implications depend critically on the institutional context in which decentralization operates (Chen et al., 2026). The local governance literature emphasizes that the effects of fiscal decentralization are mediated by vertical accountability mechanisms, local administrative capacity , and the degree of policy discretion granted to subnational governments (Bardhan and Mookherjee, 2006; Wang et al., 2025). Therefore, based on the current system, there is an obvious logical break in the study of the direct impact of fiscal decentralization on technological innovation of enterprises. This paper focuses on the mechanism of Chinese fiscal decentralization on technological innovation of enterprises and the characteristics of property rights dependence through the construction of a moderated intermediation model .  ……  **6.2 \| Policy Implications**  In his article “Adherence to Historical Materialism Continuously Opens New Frontiers for the Development of Marxism in Contemporary China”, General Secretary Xi Jinping, for the first time, juxtaposed the roles and counterproductive effects between the productive forces and the relations of production, emphasizing that “the productivity criterion must be understood comprehensively and accurately, and cannot be absolutized, and the productivity criterion must not be understood by setting aside the relations of production and superstructure” . Fiscal decentralization belongs to the scope of the relations of production, how to realize the synergy of interests and convergence of objectives of all the main parties in the Chinese fiscal decentralization and its embedded “central government-local government-enterprise” three-layer commission-agent relationship, is the key to promote technological innovation of enterprises to form a new quality of productivity. This is the key to promoting technological innovation and forming new quality productivity. This pattern is consistent with the political competition mechanism documented by Yu et al. (2022), who show that Chinese local governments engage in strategic behavior aimed at improving their relative performance evaluations. Supporting actions favored by the central government allows local authorities to signal compliance and competence, thereby enhancing their position in the promotion tournament. | |
| **Valuable comment 4**  Including a table that lists the names and descriptions of the variables would enhance navigation throughout the article. | Thank you for this helpful suggestion. In response, we have added a new table listing the names, definitions, and descriptions of all variables used in the analysis (Please see P.10). This table is intended to improve the clarity and navigability of the manuscript and to facilitate readers’ understanding of the empirical setup. |
| **The following contents in red are added, supplemented, or revised in the context of this paper** **3.1.5 \| Control Variables** The control variables in this paper are selected from the enterprise level and the regional level respectively. The control variables at the enterprise level are: equity concentration ($\text{Herf}$), measured by the sum of squares of the shareholding ratios of the top five shareholders; cash holdings ($\text{Cash}$), measured by the ratio of money funds to total assets; company size ($\text{Size}$) measured by the natural logarithm of total assets; debt-to-asset ratio ($\text{Debt}$), measured by the ratio of liabilities to total assets; and the age of company listing ($\text{Age}$), measured by the ratio between the year of attribution of the panel data and the age of private companies. , measured using the natural logarithm of the difference between the year of attribution and the year of listing of the panel data. Regional level control variables: regional economic development level ($\text{GDP}$), measured by GDP per capital; openness level ($\text{FDI}$), measured by FDI per capital; degree of financial support ($\text{Finance}$), measured by the ratio of value added of the financial sector to GDP; and industry development level ($\text{Industry}$), measured by the ratio of the value-added of the secondary sector in GDP. The names and descriptions of the variables are shown in Table 1.  **TABLE 1 \| Variables and definitions table.** **Variable type** \| **Variables** \| **Name** \| **Description**; Explained Variable \| $\text{Innovation}$ \| Enterprise technological innovation \| The number of patent applications; Explanatory Variable \| $\text{Fisdec}$ \| Fiscal decentralization \| The fiscal autonomy indicator; Mediating Variable \| $\text{Prefer}$ \| Local government innovation preference \| Local fiscal science and technology expenditures / local fiscal general budget expenditures; Moderating Variable \| $\text{State}$ \| Nature of property rights \| 1 represents state-owned enterprises, 0 represents private enterprises.; Control Variable \| $\text{Herf}$ \| Equity concentration \| The sum of squares of the shareholding ratios of the top five shareholders; \| $\text{Cash}$ \| Cash holdings \| Money funds / total assets; \| $\text{Size}$ \| Company size \| The natural logarithm of total assets; \| $\text{Debt}$ \| Debt-to-asset ratio \| Liabilities / total assets; \| $\text{Age}$ \| Age of company listing \| ln(the year of attribution-the age of private companies）; \| $\text{GDP}$ \| Regional economic development level \| GDP per capital; \| $\text{FDI}$ \| Openness level \| FDI per capital; \| $\text{Finance}$ \| Degree of financial support \| Value added of the financial sector / GDP; \| $\text{Industry}$ \| Industry development level \| Value-added of the secondary sector / GDP | |
| **Valuable comment 5**  The statistics table should present the data before logarithmization, as it is important and interesting to see for example the actual number of patents filed by individual companies and the extent of variation. If I calculated correctly, the maximum is about 800 patents per year, which is a significant figure. It may be worth exploring what type of company is responsible for such a remarkable level of innovation. | Thank you for this valuable suggestion regarding the presentation of descriptive statistics.  To this end, we have revised the manuscript to supplement the descriptive statistical results based on the raw number of patent applications (Please see P.11-12).  As the reviewer has accurately pointed out, the distribution of patent application data is significantly right-skewed—in a given year, only a small number of firms file an exceptionally large number of patents. This characteristic not only highlights the outstanding innovation capacity of leading enterprises but also confirms the necessity of applying logarithmic transformation in the empirical analysis: this method can effectively mitigate the interference of extreme values and ensure the robustness of estimation results. |
| **The following contents in red are added, supplemented, or revised in the context of this paper** **3.4 \| Descriptive Statistics of Main Variables** The descriptive statistics of the main variables in this section are shown in the table below. From the Table 2, it can be seen that among the 9,180 companies/year samples, the mean value of technological innovation of enterprises (measured by the number of patent applications) stands at 107.918, with a median value of 82. This indicates a distinct right-skewed distribution of the sample, where more than half of the firms exhibit innovation levels below the sample average. The standard deviation of corporate technological innovation is 203.622, with a minimum value of 1 and a maximum value of 4,502. These figures further reflect the significant heterogeneity and high degree of dispersion in technological innovation outputs across firms. Following this logic, further found that more than 50% of Chinese provinces (including municipalities directly under the central government) have higher-than-average degrees of fiscal decentralization and local government innovation preferences, but there are large differences in the degree of fiscal decentralization and local government innovation preferences among provinces (including municipalities directly under the central government). **TABLE 2 \|** Descriptive statistics table. **Variables** \| **Observed value** \| **Mean** \| **Standard deviation** \| **Minimum value** \| **Maximum value** \| **Median**; $\text{Innovation}$ \| 9180 \| 107.918 \| 203.622 \| 1 \| 4502 \| 82; $\text{Fisdec}$ \| 9180 \| 0.649 \| 0.187 \| 0.251 \| 0.926 \| 0.714; $\text{Prefer}$ \| 9180 \| 0.034 \| 0.017 \| 0.008 \| 0.068 \| 0.038; $\text{Herf}$ \| 9180 \| 0.147 \| 0.107 \| 0.017 \| 0.533 \| 0.115; $\text{Cas}\text{h}$ \| 9180 \| 0.168 \| 0.112 \| 0.019 \| 0.560 \| 0.139; $\text{Debt}$ \| 9180 \| 0.437 \| 0.199 \| 0.060 \| 0.929 \| 0.433; $\text{Age}$ \| 9180 \| 2.336 \| 0.593 \| 0.768 \| 3.273 \| 2.381; $\text{Size}$ \| 9180 \| 22.378 \| 1.218 \| 20.134 \| 26.080 \| 22.218; $\text{GDP}$ \| 9180 \| 11.131 \| 0.453 \| 10.184 \| 12.013 \| 11.146; $\text{FDI}$ \| 9180 \| 8.456 \| 1.200 \| 5.825 \| 10.580 \| 8.569; $\text{Finance}$ \| 9180 \| 0.083 \| 0.040 \| 0.034 \| 0.199 \| 0.071; $\text{Industry}$ \| 9180 \| 0.531 \| 0.117 \| 0.359 \| 0.839 \| 0.503; $\text{State}$ \| 9180 \| 0.406 \| 0.491 \| 0 \| 1 \| 0 | |
| **Valuable comments from Reviewer #2:** | |
| **Valuable comment 1**  My first concern is that while the manuscript invokes a three-tier “principal-agent” framework, the conceptual clarity is lacking. Theoretical underpinnings (i.e., linkage between fiscal decentralization and innovation suppression) are insufficiently grounded in local governance literature. The authors mostly cite economic literature, overlooking well-established debates in public administration and fiscal federalism regarding vertical accountability, local autonomy, and institutional capacity. The author may enhance the theoretical framework by integrating key works in local governance and decentralization theory. Please also consider how institutional quality, local political capacity, and discretion mediate the impact of decentralization on policy outcomes. | We thank the reviewer for this important comment. We agree that the explanatory power of the three-tier principal–agent framework depends on its institutional grounding. In the revised manuscript, we have strengthened the theoretical foundation by integrating insights from the local governance and fiscal federalism literature, particularly regarding vertical accountability, local autonomy, and institutional capacity (Please see P.5).  Specifically, we clarify that the innovation-inhibiting effects of fiscal decentralization are not mechanical but are mediated by governance capacity and policy discretion at the local level. We also explicitly acknowledge that decentralization may yield different outcomes under alternative institutional conditions, thereby refining the scope of our theoretical claims. |
| **The following contents in red are added, supplemented, or revised in the context of this paper**  **2 \| Theoretical Analysis and Research Assumptions**  From the perspective of the evolution path of China’s fiscal system, before the 1994 tax-sharing reform, the fiscal system mainly acts directly on microeconomic subjects through embedding enterprises (especially state-owned enterprises). The tax-sharing reform has embedded China's finances in the economic cycle, forming a three-tier principal-agent relationship among the central government, local governments and enterprises. Local governments play a pivotal role in connecting the central government and enterprises in their jurisdictions. While the three-tier principal–agent framework provides a useful analytical lens, its implications depend critically on the institutional context in which decentralization operates (Chen et al., 2026). The local governance literature emphasizes that the effects of fiscal decentralization are mediated by vertical accountability mechanisms, local administrative capacity , and the degree of policy discretion granted to subnational governments (Bardhan and Mookherjee, 2006; Wang et al., 2025). Therefore, based on the current system, there is an obvious logical break in the study of the direct impact of fiscal decentralization on technological innovation of enterprises. This paper focuses on the mechanism of Chinese fiscal decentralization on technological innovation of enterprises and the characteristics of property rights dependence through the construction of a moderated intermediation model .  **2.1 \| Fiscal Decentralization and Technological Innovation of Enterprises**  As an important institutional arrangement to adjust the allocation of financial resources between the central government and the local government, fiscal decentralization guides enterprises in technological innovation by enhancing the local government’s support for science and technology (Pu et al. 2023). However, due to the high uncertainty of technological innovation, it is worth pondering whether fiscal decentralization necessarily promotes technological innovation of enterprises. Enterprises may face the following problems in the process of carrying out technological innovation activities: firstly, technological innovation is a trial-and-error process, and the marginal productivity of R&D investment in the pre-innovation period is low, so it is difficult for enterprises to obtain substantial output returns, which will generate negative feedback on the subsequent development of innovation activities (Fu and Qian, 2023). Second, technological innovation is the exploration of the feasibility of new technologies. In order to ensure the profitability of technological innovation and set up innovation barriers to prevent potential competitors from entering, enterprises need to continuously track the technological frontier, but they may fall into the capability trap of “over-exploration” (Acemoglu et al. 2022). Finally, in an era characterized by exponential technological advances, firms may have the problem of underestimating the rate of technological obsolescence. In the era of knowledge economy, the speed of technological update is too fast, and it is often difficult for enterprises to accurately judge when to change or stop their investment strategy in technological innovation, and thus they are more inclined to increase their R&D investment in existing technologies, which is not conducive to the output of high-quality technological innovation results. Furthermore, in settings characterized by strong vertical accountability but limited local administrative capacity, decentralization may incentivize local governments to prioritize short-term, measurable outcomes over long-term innovation investments, which are inherently uncertain and difficult to evaluate (Fang et al., 2025; Wang et al., 2025).  Wang et al. (2024) and Xie and Wu (2024) found that enterprises in the growth and decline stages focus on “survival”, while enterprises in the maturity stage focus on “development”. Mature enterprises tend to invest in innovative projects with high capital investment and long payback period but high expected returns (Israel and Sali, 2025). Therefore, financial subsidies and tax incentives mainly have an incentive effect on the technological innovation behavior of enterprises in the mature stage of the life cycle, and have a smaller incentive effect on the technological innovation of enterprises in the growth and decline stages. In addition to the time dimension (enterprise life cycle stage), there is also significant heterogeneity in the innovation policy incentive effects in the cross-sectional dimensions of industry attributes (Zhong et al. 2022), enterprise size (Xiang et al. 2025), and the degree of market competition (Holmstrom and Milgrom, 1991). However, local governments often implement “one-size-fits-all” policy design, ignoring the differences and autonomous selectivity of enterprises, and even directly “circle the winners” with selective and non-universal policies, thus failing to form effective incentives for technological innovation. Comprehensive analysis of the above dual perspective of the enterprise and the government, put forward the following hypothesis:  Hypothesis H1: fiscal decentralization has a significant inhibitory effect on enterprise technological innovation. | |
| **Valuable comment 2**  Second, the use of “local government innovation preference” as a mediating variable is both central and problematic. Its operationalization via the ratio of science & tech expenditure to general budget lacks depth and does not convincingly reflect "preference"—a psychological and political orientation—especially when interpreted as a mediating institutional mechanism. | We thank the reviewer for raising this important conceptual issue. We respectfully clarify that in our study, local government innovation preference is not intended to capture a psychological inclination of individual policymakers. Rather, it is defined as an institutionalized and revealed policy orientation manifested through fiscal allocation decisions under resource and governance constraints.  We have revised the manuscript (Please see P.9) to clarify this conceptualization and to strengthen the theoretical justification for using science and technology expenditure intensity as a measure of local government innovation preference. We also acknowledge the scope and limitations of this proxy while maintaining its appropriateness for capturing policy-relevant orientation in a highly institutionalized budgeting system. |
| **The following contents in red are added, supplemented, or revised in the context of this paper**  **2.2 \| The mechanism of fiscal decentralization on enterprise technological innovation and its heterogeneity**  **2.2.1** **\| Fiscal decentralization and local government innovation preference**  China’s first economic decentralization was launched in 1958, and although it did not achieve the expected results due to the impact of the Great Leap Forward, the large-scale multi-dimensional decentralization objectively constituted a kind of institutional experiment (Pu et al. 2023), providing a blueprint for subsequent reforms in a path-dependent manner. 1994 saw the nationwide implementation of the tax-sharing reform, which marked the beginning of the fiscal decentralization based on standardized tax divisions. the formation of a fiscal decentralization system based on a standardized tax division, and local governments have since gained relatively stable and independent economic autonomy. Unlike economic decentralization, according to the formal institutional expression of the constitution, China is still a typical centralized unitary state. Its main features are: local government power is delegated by the central government; provincial and non-provincial key officials are appointed by the central government, and other local officials are appointed from the top down; the hierarchical power matrix between the different levels of government, and vertical inter-governmental interactions follow a “command-and-obey” pattern of behavior. In China’s hierarchical governance system, local officials are embedded in a promotion tournament characterized by relative performance evaluation rather than electoral accountability (Fang et al., 2025). Promotion prospects depend critically on how well local leaders perform relative to their political peers, especially in terms of indicators emphasized by the central government. As shown by Yu et al. (2022), this political competition generates strong strategic interactions among local governments, particularly among politically proximate jurisdictions, and induces local officials to align their policy actions with central priorities in order to accumulate “points” in the promotion tournament.  Therefore, the process of economic decentralization in China is also the process of local governments accepting the central government’s performance appraisal and carrying out “political championships” (Yang et al. 2025). The government is a typical multitasking political organization, in which the local government, as an agent, needs to trade-off between multiple potentially conflicting tasks, while the central government, as a principal, needs to guide the local government to rationally allocate its attention and effort among multiple tasks through the design of an optimal incentive contract (Li et al., 2025). For a long time, the central government has mainly used simple and measurable relative GDP growth indicators to assess the performance of local officials, resulting in a distorted allocation of local governments’ efforts among multiple tasks (Hall and Maffioli, 2008), and creating a “crowding out effect” on technological innovation. “the proportion of the added value of core industries in the digital economy to GDP” and other technological innovation indicators have been successively incorporated into the government’s work objectives, while the GDP growth rate indicator continues to be downwardly adjusted or even blurred (qualitative expression). By changing the relative incentive strengths of economic growth and technological innovation, the central government has corrected the distortion of local governments' attention and efforts in the multitasking political organization, and guided local governments to shift from “competing for growth” to “competing for innovation” (Wang et al. 2025) . In this study, local government innovation preference is conceptualized as an institutionalized policy orientation revealed through fiscal allocation decisions, rather than a psychological inclination of individual policymakers.  ……  **3.1.3 \| Mediating Variable**  The mediating variable in this paper is local government innovation preference ($\text{Prefer}$)，which is measured by the ratio of local fiscal science and technology expenditures to local fiscal general budget expenditures, with reference to the practice of Ye and Zeng (2024). The primary approaches through which governments support innovation activities include establishing institutional mechanisms to protect innovation, developing hardware and software environments, and providing direct fiscal support. The proportion of science and technology expenditure in total government fiscal expenditure reflects the degree of governmental participation in regional innovation initiatives. It represents tangible policy commitments and resource allocation decisions of the government, and exerts a more practical impact on enterprises’ innovation activities. | |
| **Valuable comment 3**  Third, the author employs the interaction between geographic distance to Beijing and a time trend as an instrument for fiscal decentralization. While this aligns with some spatial attenuation logic, the justification for exclusion restriction remains weak. Geographic distance could plausibly correlate with regional developmental trajectories, market access, and innovation potential. This undermine the exogeneity assumption. | We thank the reviewer for this important comment regarding the exclusion restriction of our instrumental variable. We agree that geographic distance to Beijing could plausibly correlate with regional development trajectories, market access, and innovation potential.  In the revised manuscript, we have substantially clarified the institutional logic underlying our instrument and softened overly strong exogeneity claims (Please see P.13-14). Specifically, we emphasize that distance to Beijing reflects administrative and political distance within China’s centralized fiscal system rather than proximity to markets or innovation centers. Beijing is neither China’s primary commercial hub nor its main innovation cluster, which alleviates concerns related to market access and inherent innovation potential.  We further clarify that our identification relies on the interaction between geographic distance and temporal changes in fiscal governance, rather than on distance per se. Time-invariant regional characteristics correlated with distance are thus differenced out, and any remaining distance-related effects are unlikely to vary over time in a way that systematically affects fiscal decentralization but not firm innovation through the institutional channel of interest.  We have revised Section 4.2.3 accordingly, elaborating on how the empirical design of this study mitigates the aforementioned concerns. We hope that our revisions will meet with your approval. |
| **The following contents in red are added, supplemented, or revised in the context of this paper** **4.2.2 \| Other Robustness Tests** To test whether the baseline regression results are susceptible to other potential biases and identification concerns, this paper conducts a series of robustness tests, with the regression results presented in Table 4. First, considering that firm-level innovation activities may exhibit intertemporal serial correlation and that unobserved firm-specific heterogeneous characteristics may persist across years, this paper clusters standard errors at the firm level. This adjustment can control for the problem of within-firm dependence and ensure that statistical inference will not be biased due to underestimated standard errors, with the corresponding regression results shown in Table 4, column (1). Second, to address the potential issue that unobservable region-specific time-varying factors may affect both fiscal decentralization and firm innovation simultaneously, this paper further controls for region × year fixed effects. This model specification can absorb all time-varying region-specific heterogeneous shocks, including differentiated regional development trajectories, innovation policy cycles, and macroeconomic fluctuations, thereby allowing firms in different regions to respond heterogeneously to common time shocks. The estimation results after incorporating region × year fixed effects are presented in Table 4, column (2). Finally, given the promulgation of relevant policies in China in 2016, which marked a major institutional adjustment in central-local fiscal relations, the behaviors of firms and local governments in that year may have reflected more transitional responses rather than stable policy effects. To avoid potential interference caused by such transitional dynamics, this paper excludes the sample observations of 2016 and re-estimates the model, with the relevant results shown in Table 4, column (3). Across all model specifications, the regression coefficients of fiscal decentralization remain significantly negative at the 1% statistical level, which is consistent with the results of the benchmark regression, indicating that the findings of the benchmark regression in this paper are robust. **TABLE 4 \|** Other robustness test results. **Variables** \| **(1)** \| **(2)** \| **(3)**; $\text{Fisdec}$ \| -1.253^***^(0.201) \| -16.110^***^(2.685) \| -1.278^***^(0.107); $\text{Herf}$ \| -0.047 (0.223) \| -0.080 (0.218) \| -0.024 (0.108); $\text{Cas}\text{h}$ \| -0.159 (0.099) \| -0.193^(**) (^0.098) \| -0.173^(***) (^0.063); $\text{Size}$ \| 0.087^***^(0.029) \| 0.0837^***^(0.027) \| 0.0845^***^(0.013); $\text{Debt}$ \| -0.073 (0.083) \| -0.005 (0.083) \| -0.072 (0.048); $\text{Age}$ \| 0.437^***^(0.059) \| 0.151^**^(0.072) \| 0.417^***^(0.029); $\text{GDP}$ \| 0.823^***^(0.107) \| 4.285 (3.233) \| 0.852^***^(0.063); $\text{FDI}$ \| -0.012 (0.030) \| 2.227^***^(0.783) \| 0.005 (0.019); $\text{Finance}$ \| -0.698 (1.156) \| 86.45 ^***^(21.850) \| -0.745 (0.603); $\text{Industry}$ \| -0.0985 (0.573) \| -51.75^***^(17.570) \| -0.270 (0.267); $\text{Constant}$ \| -6.640^***^(1.089) \| -33.260 (41.050) \| -6.906^***^(0.581); *R*^2^ \| 0.509 \| 0.573 \| 0.519; *N* \| 9 180 \| 9 180 \| 8 160 Note: ^*,**and***^denote significance at the 10%, 5% and 1% levels, respectively; robust standard errors for clustering are in parentheses in column (1), and robust standard errors in parentheses in column (2) and column (3). **4.2.3 \| Endogeneity Test** Omitted variables, measurement error of fiscal decentralization, and possible reverse causality between fiscal decentralization and firms’ technological innovation can affect the empirical results. To mitigate the above endogeneity problems, this paper uses the interaction term between the geographic distance ($\text{Distance}$) and the time trend ($\text{Year}$) from the capital city of each province to Beijing (the central government) as an instrumental variable for fiscal decentralization. Fiscal decentralization is not only the result of top-down institutional design, but also due to the “spatial attenuation” of public goods supply capacity and the local government’s advantage of being located at (Wang et al., 2025; Song et al., 2025). The geographical distance to Beijing mainly reflects attributes of administrative and political distance: The geographical distance to Beijing mainly reflects attributes of administrative and political distance: the farther the local government is from Beijing, the higher the degree of fiscal decentralization it obtains, which satisfies the relevance requirement of instrumental variables. Meanwhile, as a natural factor, geographical distance also meets the exogeneity requirement of instrumental variables. This is because Beijing is not China’s core commercial or technological innovation hub, and major domestic innovation centers are geographically distant from Beijing, meaning this distance does not directly affect firms’ technological innovation. However, using only the indicator of geographical distance between each provincial capital and Beijing would result in the loss of time-varying information. Therefore, this paper introduces a time trend term. Time-invariant regional development characteristics are absorbed by regional fixed effects, while time-varying regional shocks are controlled by the interaction term of geographical distance between each provincial capital and Beijing × year fixed effects. This interaction term is then employed as the final instrumental variable.  The endogeneity test results show that the first stage F-statistic is greater than 10 and significant at the 1% level, which can reject the original hypothesis of weak instrumental variables, indicating that the interaction term between the geographic distance from provincial capital cities to Beijing ($\text{Distance}$) and the time trend ($\text{Year}$) is not a weak instrumental variable and meets the requirement of correlation. After the introduction of instrumental variables, the sign (i.e., direction) of the regression coefficient of fiscal decentralization (-4.163) is consistent with the baseline regression results and passes the significance test at the 1% level. The above results indicate that the inhibitory effect of fiscal decentralization on firms' technological innovation still holds after mitigating potential endogeneity. **TABLE 5 \|** Endogeneity test results. **Variable** \| **2SLS**; $\text{Distance}\text{*}\text{Year}$ \| -4.163 ^***^(0.238); $\text{Herf}$ \| -0.046 (0.098); $\text{Cas}\text{h}$ \| -0.282 ^***^(0.057); $\text{Size}$ \| 0.069^***^(0.011); $\text{Debt}$ \| -0.001 (0.044); $\text{Age}$ \| -0.120^***^(0.036); $\text{GDP}$ \| 0.780^***^(0.058); $\text{FDI}$ \| -0.034^**^(0.017); $\text{Finance}$ \| 1.438^**^(0.583); $\text{Industry}$ \| 0.288 (0.246); Firm Fixed Effects \| Yes; Year fixed effects \| Yes; $\text{Constant}$ \| -6.463 ^***^(0.610); $\text{R}^{\text{2}}$ \| 0.944; $\text{N}$ \| 9 180; F-statistic for the first period \| 1 661.820^***^ Note: ^*,**and***^denote significance at the 10%, 5% and 1% levels, respectively; robust standard errors are in parentheses. | |
| Fouth, the manuscript idealizes SOEs as effective agents of innovation without sufficient critical reflection. It claims SOEs overcome “market failure” via dual coordination mechanisms but downplays inefficiencies, rent seeking, or governance challenges associated with SOEs in China and elsewhere. Please balance the discussion with references to literature on SOE inefficiencies and performance heterogeneity. | Thank you for this important comment. We agree that the role of SOEs in innovation activities should not be simply portrayed as inherently superior or universally efficient.  To this end, we have revised the manuscript to provide a more balanced and critical discussion of SOEs (Please see P.7). Specifically, we cite relevant literature to point out that SOEs face various issues including soft budget constraints, rent-seeking behavior, and governance-related problems, all of which may undermine innovation efficiency. Meanwhile, we clarify that under specific institutional and governance conditions, market-oriented reforms may help SOEs partially mitigate certain market and government failures. Nevertheless, such reform arrangements cannot eradicate government failures, nor can they eliminate the efficiency losses associated with the nature of state ownership. |
| **The following contents in red are added, supplemented, or revised in the context of this paper**  **2.2.2 \| Local government innovation preference and enterprise technological innovation and property rights heterogeneity**  Technological innovation has the attribute of public goods, and the private benefits that innovation subjects can obtain from it are lower than the social benefits, resulting in the output and efficiency of enterprise innovation not reaching the optimal level. In order to overcome the “market failure” caused by the positive externalities of technological innovation, the government may need to increase its fiscal expenditure on science and technology and make concerted use of policy tools such as financial subsidies and tax incentives to motivate enterprises to strengthen technological innovation. Because of the complexity of the behavioral responses of enterprises in economies in transition, local government innovation incentives may promote substantive technological innovation or become a strategic tool for enterprises to extract policy resources.  The existing literature generally follows the analytical framework of neoclassical economics and treats State-owned enterprises as general market players, arguing that the expansion of State-owned property rights is inefficient in areas that require sustained innovation (Li et al. 2025). In practice, however, SOEs have been an important tool in many countries around the world to address “market failures” caused by private returns falling short of social returns. Emerging economies in Latin America, such as Argentina, Brazil, Chile, Colombia, Mexico, etc (Hall & Maffioli, 2008) and developed economies, such as France, Norway, Austria, etc. (Chang et al., 2002) have used SOEs as a way to channel the development of basic research or high-risk emerging industries to cope with the shortage of innovation supply. In China, SOEs have also replaced state-led research institutes and higher education institutions as the new “technology diffusion centers” (He et al., 2023). The fundamental reason why SOEs play an important role in technological innovation is that, as the core institutional arrangement of the socialist market economic system (Wang and Yu, 2025), SOEs, in addition to the pursuit of profits in the sense of financial accounting, the national rent, i.e., the national mission, is also an important goal of their behavior. At the same time, extensive evidence suggests that SOEs are often associated with inefficiencies, soft budget constraints, rent-seeking behavior, and governance challenges, which may undermine innovation efficiency if not properly addressed. Although the non-market contractual characteristics of SOEs are a great dissolution of the error correction function of the market mechanism (Bagwell and Ramey, 1996), through a series of sustained market-oriented reforms such as the mixed ownership reform and the reform of dividend rights incentives, SOEs can combine the advantages of the market and the government’s two modes of economic coordination to partially mitigate certain forms of market failure and government failure under specific institutional and governance conditions. However, such institutional arrangements cannot eliminate government failure, nor can they avoid the efficiency losses associated with the nature of state-owned property rights. As an institutional trade-off, the innovation performance of SOEs is closely tied to the quality of governance and the depth of reforms.  Unlike SOEs, a significant portion of private enterprises are not motivated by the local government’s preference for innovation, but rather by access to government financial resources to ease financing constraints and operational pressures. An important consequence is that the policy arbitrage behavior of private enterprises forms a de facto “political asylum”, which in turn interferes with their technological innovation decisions. According to the theory of strategic blocking, as long as there are long-term profits, incumbent firms may be able to block the entry of potential competitors through technological innovation (Bagwell, 1990). However, the “political patronage” provided by the government in terms of finance (S&T spending, government subsidies, tax incentives), land, credit, market access (investment restrictions, project approval rights, business licensing rights), etc. can hedge against the competitive pressure in the market and reduce the threat of entry of incumbent firms, so that the policy arbitrage becomes a “viable” alternative for the incumbent firms’ technological innovation deterrence, which is a “viable” alternative for the incumbent firms. A “viable” alternative (Lee and Lee, 2022; Szczygielski et al. 2017). According to the logic of collective action, in an innovation race between multiple incumbents and potential entrants, each incumbent wants to enjoy the benefits of the others without paying the costs (i.e., “free-riding”), resulting in the higher the barriers to entry, the lower the incentives for firms to technologically innovate (Wang et al. 2020).  Accordingly, this paper argues that the role of SOEs in the field of innovation is conditional and context-dependent, rather than an indication of their inherent superiority over private enterprises. Integrating the analysis of the mechanism of fiscal decentralization on the role of enterprise technological innovation and property rights heterogeneity, the following hypotheses are proposed:  Hypothesis H2: Local government innovation preference has a mediating role between fiscal decentralization and enterprise technological innovation, and fiscal decentralization indirectly affects enterprise technological innovation by influencing local government innovation preference.  Hypothesis H3: The mediating role of local government innovation preference is characterized by property rights dependence, and fiscal decentralization promotes (inhibits) technological innovation of state-owned (private) enterprises through influencing local government innovation preference. | |
| We greatly appreciate your effort in reviewing this paper and your treasured comments provided for our paper, all of which would indeed help us to learn how to write a top-tier academic paper and how to enhance our manuscript. Although we have to make efforts to revise this paper, we indeed learn a lot. We are really appreciated your valuable comments and suggestions. Thank you so much. | |
