# Supplementary material for: Fiscal decentralization, local government innovation preference, and enterprise technological innovation: Evidence from China
Source: PLoS One. 2026 Apr 15;21(4):e0347025. doi: 10.1371/journal.pone.0347025 (PMC13082718; doi:10.1371/journal.pone.0347025)
Supplement: Attachment — Submitted filename: Responses_to_Reviewers_auresp_2.docx [file pone.0347025.s004.docx]

**Detailed Responses to Editor and Reviewers**

**Dear Editor and Reviewers,**

We are glad and grateful for the opportunity to improve our manuscript’s quality. We much appreciate your constructive comments, intellectually stimulating suggestions, and encouraging words. We have carefully considered all the comments and revised our manuscript accordingly and the changes and revisions made in this manuscript are marked in RED. Thank you for providing valuable and insightful comments, which indeed helped us to learn how to write a top-tier academic paper and how to enhance our manuscript. Thank you so much.

| **Valuable comments from Reviewer #1:** | |
| --- | --- |
| **Valuable comment 1**  For example, I wonder if the logic presented in the introduction about the negative impact of decentralization on innovation under the Chinese model of decentralization is valid. (lines 85-89) After all, if we have a system in which local authorities strive to obtain "points" from the state, they will decide to implement innovative activities as long as they are consistent with state policy. Moreover, they will decide to implement such activities even against the needs of residents – because it is not their needs, but the center's recommendations that are being implemented in such circumstances. Moreover, it is precisely this logic that the authors demonstrate in their conclusion. | **Response to valuable comment 1**  We greatly appreciate your comment that our discussion of the negative impact of China’s fiscal decentralization on innovation remains insufficient. Accordingly, we have supplemented relevant arguments on how decentralization inhibits innovation in the manuscript (Please see P.3 and P.5).  Specifically, under China’s decentralization model, it is true that—as you pointed out—local authorities will prioritize innovation activities as long as they meet the criteria for officials to gain performance points. However, innovation activities are characterized by large investment scale, high risk, and vulnerability to imitation and infringement. The scientific and technological innovation output in one region can promote the economic and social development of other regions in a short period. Such positive externalities often lead local governments to lack incentives for innovation and tend to reduce government spending on science and technology.  Under the current performance evaluation system, local governments are constrained by limited time, effort, and resources, making it difficult to simultaneously achieve multiple goals such as economic growth and innovation-driven development. This results in selective implementation and goal substitution, where local officials prioritize short‑term visible GDP growth over long‑term investment in innovation. |
| **The following contents in red are added, supplemented, or revised in the context of this paper**  **1 \| Introduction**  Under the background of diminishing marginal effect of monetary policy, the importance of finance for economic development and scientific and technological innovation has become the consensus of governments. As a national fundamental institutional arrangement of fiscal power distribution relationship [1], the in fiscal decentralization China implies a three-layer principal-agent relationship among the central government, local governments and enterprises [2,3], which constitutes a de facto institutional constraint on the technological innovations of the enterprises through the government’s “tangible hand”. Second, due to differences in political and economic environments, fiscal decentralization practices vary across countries. In China, fiscal decentralization has played a particularly important role [4]. The coexistence of political centralization and economic decentralization ensures central government control while aligning local government incentives with market economic development [5,6]. Decentralization reforms require sufficient institutional and state capacity [4]. In Russia, the central government has gradually lost leadership during decentralization and failed to provide sufficient incentives for local development [7]. In many developing countries, decentralization is associated with insufficient public resource supply and increased inequalit. Against this backdrop, examining China’s local and county fiscal system provides practical experience for other centrally planned countries facing decentralization challenges.  This paper analyzes the existing literature from the technical innovation effect of fiscal decentralization and the influence factors of enterprise technological innovation in two aspects: First, the technical innovation effect of fiscal decentralization. The effect of fiscal decentralization on technological innovation is mainly analyzed by provincial and municipal local governments [8], and there are two distinct views: “decentralization inhibition theory” and “decentralization promotion theory” [9]. Scholars who support the “decentralization promotion theory” believe that fiscal decentralization is conducive to strengthening the government’s innovation function [10], and promoting the development of technological innovation activities of local governments and their efficiency [11]. Scholars who support the “decentralization inhibition theory” believe that fiscal decentralization will exacerbate the vicious competition between governments and make the investment structure of local governments show the obvious self-interested characteristic of “light on innovation and heavy on production” [9,12,13]. This inhibitory effect does not stem from decentralization itself, but rather from the promotion tournament mechanism embedded in China’s hierarchical governance system. When local officials are constrained by limited time, energy, and resources, and their career advancement depends heavily on relative performance evaluation set by higher authorities—especially GDP growth—they tend to prioritize short-term, quantifiable productive investments over innovation inputs characterized by high uncertainty and long cycles [14,15]. However, when the central government adjusts the evaluation criteria and strengthens innovation indicators, the same fiscal decentralization system can instead incentivize local governments to engage in an innovation tournament [1].The reason why fiscal decentralization reduces the local government’s innovation preference is that, on the one hand, political competition among Chinese local officials is structured as a performance-based tournament overseen by higher-level authorities. Yu et al. provide systematic evidence that such competition leads to spatial interactions among politically connected jurisdictions, as local officials strategically adjust their behavior in response to their peers in order to improve their relative standing in the promotion hierarchy [14,15]. Compared with the productive financial expenditures such as infrastructure, the innovative expenditures have the attribute of public goods, and this kind of behavior of “doing others a favor” is not in line with the interests of the local government. On the other hand, scientific and technological innovation is highly uncertain. Under the promotion championship mechanism of political centralization and economic decentralization, local government officials with the attribute of “politicians” tend to squeeze out the expenditures on unproductive public goods such as innovation in pursuit of economic growth during their tenure [12].  ……  **2.1 \| Fiscal Decentralization and Technological Innovation of Enterprises**  As an important institutional arrangement to adjust the allocation of financial resources between the central government and the local government, fiscal decentralization guides enterprises in technological innovation by enhancing the local government’s support for science and technology [10]. Fiscal decentralization endows local governments with fiscal autonomy and information advantages, which in theory can support the implementation of locally tailored innovation policies [10,11]. However, under the dual context of political centralization and economic decentralization in China [5,6], local officials face a multi-task principal-agent problem, and promotion incentives dominate resource allocation decisions [31]. When the performance evaluation system is heavily biased toward GDP growth, fiscal autonomy enables local governments to shift resources to infrastructure and production capacity construction that yield immediate economic returns, thereby crowding out innovation inputs. In fact, this is not contradictory to the logic that local governments carry out innovative activities to obtain “points” from the state, further indicating that the definition of such “point-scoring” behavior depends on the assessment criteria set by the central government.  However, due to the high uncertainty of technological innovation, it is worth pondering whether fiscal decentralization necessarily promotes technological innovation of enterprises. Enterprises may face the following problems in the process of carrying out technological innovation activities: firstly, technological innovation is a trial-and-error process, and the marginal productivity of R&D investment in the pre-innovation period is low, so it is difficult for enterprises to obtain substantial output returns, which will generate negative feedback on the subsequent development of innovation activities [24]. Second, technological innovation is the exploration of the feasibility of new technologies. In order to ensure the profitability of technological innovation and set up innovation barriers to prevent potential competitors from entering, enterprises need to continuously track the technological frontier, but they may fall into the capability trap of “over-exploration” [25]. Finally, in an era characterized by exponential technological advances, firms may have the problem of underestimating the rate of technological obsolescence. In the era of knowledge economy, the speed of technological update is too fast, and it is often difficult for enterprises to accurately judge when to change or stop their investment strategy in technological innovation, and thus they are more inclined to increase their R&D investment in existing technologies, which is not conducive to the output of high-quality technological innovation results. The knowledge limitations of local governments in the supply of technological innovation policies are also an important inhibiting factor for enterprise technological innovation under the current fiscal decentralization system. Furthermore, in settings characterized by strong vertical accountability but limited local administrative capacity, decentralization may incentivize local governments to prioritize short-term, measurable outcomes over long-term innovation investments, which are inherently uncertain and difficult to evaluate [1,15].  Wang et al. and Xie and Wu found that enterprises in the growth and decline stages focus on “survival”, while enterprises in the maturity stage focus on “development” [26,27]. Mature enterprises tend to invest in innovative projects with high capital investment and long payback period but high expected returns [28]. Therefore, financial subsidies and tax incentives mainly have an incentive effect on the technological innovation behavior of enterprises in the mature stage of the life cycle, and have a smaller incentive effect on the technological innovation of enterprises in the growth and decline stages. In addition to the time dimension (enterprise life cycle stage), there is also significant heterogeneity in the innovation policy incentive effects in the cross-sectional dimensions of industry attributes, enterprise size, and the degree of market competition [29,30,31]. However, local governments often implement “one-size-fits-all” policy design, ignoring the differences and autonomous selectivity of enterprises, and even directly “circle the winners” with selective and non-universal policies, thus failing to form effective incentives for technological innovation. Comprehensive analysis of the above dual perspective of the enterprise and the government, put forward the following hypothesis:  Hypothesis H1: fiscal decentralization has a significant inhibitory effect on enterprise technological innovation. | |
| **Valuable comment 2**  In the previous comments, I noted that, given the specificity of China, one should ask why its analysis might be interesting to an international audience. The only argument the authors managed to find referred to readers from other centralized countries. This is not convincing. In my opinion, it would be worthwhile, for example, to demonstrate in the conclusions whether and how these conclusions can/cannot be transferred to other countries with different decentralization systems (what other forms of support for specific actions important for the entire country can state authorities undertake in other countries, and whether this is effective – I recommend, for example, literature on decentralization and environmental protection). | **Response to valuable comment 2**  We greatly appreciate your valuable suggestion that the manuscript can be further improved in elaborating on the international relevance of studying the impact of fiscal decentralization on innovation in the Chinese context. In response, we have enriched the relevant discussion in the manuscript (Please see P.4 and P18).  Specifically, the “decentralization-inhibiting-innovation” effect revealed in this study is not unique to China, but a general outcome of the mismatch between fiscal decentralization and political incentive structures. Your advice has guided our revision. After reviewing the related literature, we find that in the field of environmental protection, cross-country studies have confirmed that decentralization may lead to a “race to the bottom”. This study extends this logic to innovation policy, showing that when local governments face competing multiple tasks and innovation is not yet included in core performance assessments, decentralization may induce short-term behavior.  This finding is informative for developing countries undertaking decentralization reforms (e.g., Indonesia, India) and federal countries seeking to optimize central-local relations (e.g., the United States, Germany). The design of decentralization needs to be aligned with central policy orientations and performance evaluation systems, rather than simply pursuing the maximization of decentralization.  In addition, China’s experience shows that selective decentralization under political centralization can stimulate innovation through central policy adjustments (e.g., the innovation-driven development strategy), thus providing complementary evidence from a non-Western context. |
| **The following contents in red are added, supplemented, or revised in the context of this paper**  **1 \| Introduction**  ……  The above literature provides ideas inspiration and paradigm borrowing for the research of this paper, but there is still room for further deepening and expanding. This paper takes A-share listed companies that regularly publish annual financial reports between 2015 and 2023 as samples, and reveals for the first time the role of fiscal decentralization in technological innovation of microeconomic agents in the three-tier principal-agent framework of “central government-local government-enterprises”. In the three-tier principal-agent framework of “central government-local government-enterprises”, this paper reveals for the first time the intrinsic mechanism of fiscal decentralization and the heterogeneity of property rights in the technological innovation of microeconomic agents. It responds to the broader discussion on decentralization and innovation under different institutional contexts, and provides China’s experience for countries with strong vertical accountability mechanisms.  Compared with the existing literature, the marginal contributions of this paper are:  (1) In terms of research perspective, it enriches and expands the academic scenario of the “central government-local government-enterprise” three-layer principal-agent model. The research on the three-tier principal-agent model of “central government-local government-enterprise” in the Chinese context is the first of its kind. The research on the three-tier principal-agent model was initiated by Nie and Zhang [22], who portrayed the equilibrium and preventive collusion contracts of the central government in the classic government-enterprise collusion phenomena such as mining accidents, environmental pollution, etc., and pointed out that the central government tends to acquiesce to the collusion of the local government and the enterprises under the decentralization (territorial management) system, compared to the centralized (vertical management) system. On the basis of analyzing and demonstrating the relationship between the central government and local governments, this paper clarifies for the first time the effect and mechanism of fiscal decentralization on technological innovation under the perspective of new quality productivity, and enriches and expands the “central government-local government-enterprise” model, which is a three-tier principal-agent model in the context of China’s major economic issues. It enriches and expands the application of the “central government-local government-enterprise” three-layer principal-agent model in China’s major real-life economic problems, which can be effectively applied to other research areas where policy outcomes are determined by tensions in central–local relations. This study identifies the boundary conditions under which fiscal decentralization affects innovation, thereby providing a more refined theoretical perspective for understanding how institutional design shapes policy implementation across national contexts.  ……  **6 \| Conclusion and Policy Implications**  **6.1 \| Research Conclusion**  Based on the panel data of A-share listed companies that regularly publish annual financial reports during the period of 2015-2023, this paper empirically examines the mechanism of Chinese-style fiscal decentralization on technological innovation of enterprises and the heterogeneity of property rights by using a moderated mediation model. The study draws the following conclusions: first, overall, fiscal decentralization has a significant inhibitory effect on corporate technological innovation, which still holds after a series of robustness tests and endogeneity tests. Second, local government innovation preference has a mediating role between fiscal decentralization and enterprise technological innovation, and fiscal decentralization indirectly affects enterprise technological innovation by influencing local government innovation preference. This is consistent with the institutional condition-dependent findings from the cross-country study by Carrillo-Pulgar et al., who show that decentralization is positively associated with environmental spending, provided that institutional quality is high [50]. This study finds that decentralization may lead to short-term behavior in environments with low institutional quality, whereas in environments with high institutional quality, decentralization can improve efficiency through informational advantages. Third, the mediating effect of local government innovation preference is characterized by significant property rights dependence, and fiscal decentralization promotes technological innovation of state-owned enterprises and inhibits technological innovation of private enterprises through influencing local government innovation preference, which proves the effectiveness of state-owned enterprises as the core institutional arrangement of the socialist market economic system.  **6.2 \| Policy Implications**  In his article “Adherence to Historical Materialism Continuously Opens New Frontiers for the Development of Marxism in Contemporary China”, General Secretary Xi Jinping, for the first time, juxtaposed the roles and counterproductive effects between the productive forces and the relations of production, emphasizing that “the productivity criterion must be understood comprehensively and accurately, and cannot be absolutized, and the productivity criterion must not be understood by setting aside the relations of production and superstructure”. Fiscal decentralization belongs to the scope of the relations of production, how to realize the synergy of interests and convergence of objectives of all the main parties in the Chinese fiscal decentralization and its embedded “central government-local government-enterprise” three-layer commission-agent relationship, is the key to promote technological innovation of enterprises to form a new quality of productivity. This is the key to promoting technological innovation and forming new quality productivity. This pattern is consistent with the political competition mechanism documented by Yu et al., in which Chinese local governments engage in various strategic behaviors to improve their standing in relative performance evaluation [14]. The nonlinear relationship between decentralization and innovation identified in this study helps local governments implement policy initiatives preferred by the central government, thereby signaling both policy compliance and governance capacity, and consolidating their advantageous position in the promotion tournament.Furthermore, the impact of fiscal decentralization on innovation exhibits property right dependence. For policymakers in other centralized systems, granting local fiscal autonomy without aligning local incentives with innovation goals may reproduce the distortion of “prioritizing production over innovation” that once occurred in China. Conversely, for federal countries seeking to strengthen central coordination, China’s experience demonstrates that performance based promotion tournaments can be effective, but also indicates that such mechanisms may induce short-term behavior if performance criteria are poorly designed. | |
| **Valuable comment 3**  Thank you for organizing the variables in the table. This makes it much easier to follow the models, and it clearly shows that the descriptions of variables that are crucial to the work's purpose are missing definitions. Specifically, how is the fiscal autonomy indicator measured – where is its definition? Similarly, I don't see a definition of the revenue decentralization indicator or the expenditure decentralization indicator. | **Response to valuable comment 3**  Thank you for your valuable suggestion regarding the clarification of variable definitions. We fully agree that clear operationalization of key variables is essential for the replicability and transparency of our empirical analysis.  In response to your comment, we have made the following revisions to the manuscript:  We have supplemented the detailed definition and measurement approach of fiscal autonomy in ***3.1.2 Explanatory Variables*** (Please see P.10). This indicator aligns with the standard approach in the fiscal federalism literature and effectively captures the extent to which local governments possess independent fiscal decision-making power. Furthermore, we have updated ***TABLE 1 Variables and Definitions*** to ensure that readers can clearly understand how this core explanatory variable is constructed.  Since the two indicators of revenue decentralization and expenditure decentralization are mainly used as alternative proxy variables for fiscal autonomy to test the robustness of the baseline regression results, rather than as core explanatory variables in the main analysis, we supplement their detailed definitions and measurement methods in ***4.2.1 Replacement of Core Explanatory Variable*** (Please see P.14) *.*  Thank you again for your constructive feedback, which has helped us improve the clarity and rigor of our empirical framework. |
| **The following contents in red are added, supplemented, or revised in the context of this paper** **1 \| Introduction** …… **3.1.2 \| Explanatory Variables** The explanatory variable in this paper is fiscal decentralization ($\text{Fisdec}$). The existing literature mainly uses four types of indicators to measure fiscal decentralization:revenue and expenditure indicator [12], fiscal autonomy indicator/fiscal self-sufficiency rate [44], incremental own revenues [45], and composite indicator [10]. An appropriate decentralization indicator needs to reflect both the inter-temporal changes in the central-local relationship and the inter-regional differences, and there is no optimal measurement method yet. In this paper, we use the fiscal autonomy indicator to portray the degree of fiscal decentralization in China from 2015 to 2023 because (1) the fiscal autonomy indicator is better than the revenue and expenditure indicator and the increment of own revenue during the study period. Fiscal autonomy, revenue and expenditure, and incremental own revenues cannot cover the entire period after the founding of New China, but the fiscal autonomy indicator is applicable to the study after the 1994 tax reform and can reflect regional differences among provinces; (2) the fiscal autonomy indicator is better than the composite indicator within the study period. Scholars tend to use a variety of measures, but different weighting indicators reflect very different facts and logic behind them, and imply completely different policy implications. For example, Zheng et al. use both revenue decentralization indicators and expenditure decentralization indicators, and find that they have opposite effects on local government S&T investment [46]. Revenue decentralization significantly inhibits local government S&T investment, while expenditure decentralization has a significant promotion effect on local government S&T investment. Therefore, various types of indicators cannot be mixed [46], and there are some problems in combining them. Lv et al. believe that the core of Chinese fiscal decentralization is fiscal revenue decentralization [47], and measure the proportion of fiscal revenue distribution among the central, provincial, municipal, and county levels, covering tax revenue, non-tax revenue, and general public budget revenue of various revenue calibers, and take it as a measurement index of fiscal decentralization. Due to the limitation of statistical data sources, the time span of fiscal decentralization data calculated directly or indirectly using this method is only from 1994 to 2014. Therefore, following the research of Jiang et al., this paper still uses fiscal autonomy, measured as the ratio of local fiscal revenue to local fiscal expenditure, as an alternative indicator of China’s fiscal decentralization [48]. Fiscal autonomy reflects the degree of discretion of local governments in formulating relevant fiscal policies. It affects the economy through local government preferences, expenditure constraints, and government bargaining power. …… **TABLE 1 \| Variables and definitions table.** **Variable type** \| **Variables** \| **Name** \| **Description**; Explained Variable \| $\text{Innovation}$ \| Enterprise technological innovation \| The number of patent applications; Explanatory Variable \| $\text{Fisdec}$ \| Fiscal decentralization \| The fiscal autonomy indicator (Local fiscal revenue / local fiscal expenditure); Mediating Variable \| $\text{Prefer}$ \| Local government innovation preference \| Local fiscal science and technology expenditures / local fiscal general budget expenditures; Moderating Variable \| $\text{State}$ \| Nature of property rights \| 1 represents state-owned enterprises, 0 represents private enterprises.; Control Variable \| $\text{Herf}$ \| Equity concentration \| The sum of squares of the shareholding ratios of the top five shareholders; \| $\text{Cas}\text{h}$ \| Cash holdings \| Money funds / total assets; \| $\text{Size}$ \| Company size \| The natural logarithm of total assets; \| $\text{Debt}$ \| Debt-to-asset ratio \| Liabilities / total assets; \| $\text{Age}$ \| Age of company listing \| ln(the year of attribution-the age of private companies）; \| $\text{GDP}$ \| Regional economic development level \| GDP per capital; \| $\text{FDI}$ \| Openness level \| FDI per capital; \| $\text{Finance}$ \| Degree of financial support \| Value added of the financial sector / GDP; \| $\text{Industry}$ \| Industry development level \| Value-added of the secondary sector / GDP …… **4.2 \| Robustness Test** **4.2.1 \| Replacement of Core Explanatory Variables** Fiscal autonomy indicator can simultaneously reflect the inter-period changes and inter-regional differences in the central-local relationship, and is a relatively better measure of fiscal decentralization, and fiscal decentralization is measured by the degree of fiscal autonomy in the benchmark regression results. Based on the study by Jin et al., fiscal revenue decentralization captures the share of own revenues of the three levels of governments (central, state/province/region and local) as a proportion of general government revenue [51]. Fiscal expenditure decentralization measures the share of spending taking place at the sub-national level (using all resources available, except borrowing) relative to total expenditure of the general government. In the benchmark regression results, fiscal decentralization is measured by the degree of fiscal autonomy. Here, the fiscal autonomy indicator is replaced by the revenue decentralization indicator and the expenditure decentralization indicator respectively to test the robustness of the benchmark regression results. Specifically, revenue decentralization is measured as the ratio of local fiscal revenue to total national fiscal revenue, while expenditure decentralization is measured as the ratio of local fiscal expenditure to total national fiscal expenditure. The regression coefficients of fiscal revenue decentralization and fiscal expenditure decentralization in columns (1) and (2) of Table 4 are both significantly negative at the 1% level, which is consistent with the benchmark regression results. | |
| **Valuable comment 4**  The authors briefly comment on the variables' statistics, but they do so in a very mechanical manner. From the perspective of the article's purpose, it would be interesting to show and discuss the differences and correlations of the most important variables, combined with detailed information. For example, does any particular region stand out in the statistics (in terms of the number of patents), or do the statistics show differences between patents filed by SOEs and private companies. Which companies (by name) are among those filing the most patents? Perhaps some industries stand out? | **Response to valuable comment 4**  Thank you for your valuable suggestions on the descriptive statistics. In response to your comments, we have substantially revised Section 3.4 Descriptive Statistics of Main Variables (Please see P.13).  The newly added stylized facts of key variables in the manuscript, especially the co-movement between regional fiscal autonomy and innovation capacity, as well as the significant divergence in innovation intensity between state-owned enterprises and private enterprises under similar fiscal environments—provide direct support for our hypothesis regarding the conditional effects of fiscal decentralization on firm innovation.  We believe these revisions have addressed your concerns and significantly strengthened the empirical foundation of this paper. |
| **The following contents in red are added, supplemented, or revised in the context of this paper** **3.4 \| Descriptive Statistics of Main Variables** The descriptive statistics of the main variables in this section are shown in the table below. From the Table 2, it can be seen that among the 9,180 companies/year samples, the mean value of technological innovation of enterprises (measured by the number of patent applications) stands at 107.918, with a median value of 82. This indicates a distinct right-skewed distribution of the sample, where more than half of the firms exhibit innovation levels below the sample average. The standard deviation of corporate technological innovation is 203.622, with a minimum value of 1 and a maximum value of 4,502. These figures further reflect the significant heterogeneity and high degree of dispersion in technological innovation outputs across firms. Following this logic, further found that more than 50% of Chinese provinces (including municipalities directly under the central government) have higher-than-average degrees of fiscal decentralization and local government innovation preferences, but there are large differences in the degree of fiscal decentralization and local government innovation preferences among provinces (including municipalities directly under the central government). **TABLE 2 \|** Descriptive statistics table. **Variables** \| **Observed value** \| **Mean** \| **Standard deviation** \| **Minimum value** \| **Maximum value** \| **Median**; $\text{Innovation}$ \| 9 180 \| 107.918 \| 203.622 \| 1 \| 4502 \| 82; $\text{Fisdec}$ \| 9 180 \| 0.649 \| 0.187 \| 0.251 \| 0.926 \| 0.714; $\text{Prefer}$ \| 9 180 \| 0.034 \| 0.017 \| 0.008 \| 0.068 \| 0.038; $\text{Herf}$ \| 9 180 \| 0.147 \| 0.107 \| 0.017 \| 0.533 \| 0.115; $\text{Cas}\text{h}$ \| 9 180 \| 0.168 \| 0.112 \| 0.019 \| 0.560 \| 0.139; $\text{Debt}$ \| 9 180 \| 0.437 \| 0.199 \| 0.060 \| 0.929 \| 0.433; $\text{Age}$ \| 9 180 \| 2.336 \| 0.593 \| 0.768 \| 3.273 \| 2.381; $\text{Size}$ \| 9 180 \| 22.378 \| 1.218 \| 20.134 \| 26.080 \| 22.218; $\text{GDP}$ \| 9 180 \| 11.131 \| 0.453 \| 10.184 \| 12.013 \| 11.146; $\text{FDI}$ \| 9 180 \| 8.456 \| 1.200 \| 5.825 \| 10.580 \| 8.569; $\text{Finance}$ \| 9 180 \| 0.083 \| 0.040 \| 0.034 \| 0.199 \| 0.071; $\text{Industry}$ \| 9 180 \| 0.531 \| 0.117 \| 0.359 \| 0.839 \| 0.503; $\text{State}$ \| 9 180 \| 0.406 \| 0.491 \| 0 \| 1 \| 0 From a regional perspective, firms in the eastern region exhibit the highest overall innovation output, with a mean value of 121.772, approximately 1.8 times that of firms in the western region (67.597) and 1.6 times that of those in the central region (74.688). This gradient is fully consistent with the distribution of fiscal autonomy: eastern provinces have the highest level of fiscal decentralization, with a mean of 0.727, followed by the central region (0.486) and the western region (0.392). From the perspective of ownership type, firm innovation performance shows significant ownership heterogeneity. The innovation intensity of SOEs is substantially higher than that of private enterprises. Consistent with the findings in Table 3, SOEs present not only a higher average innovation level but also systematically stronger innovation performance across the entire distribution. Based on Table 2, we observe that a small number of highly innovative firms dominate the overall statistics. Further analysis reveals that innovation among private enterprises is highly concentrated in a small number of high-quality firms, whereas SOEs display a more broadly distributed innovation capacity. **TABLE 3 \|** Descriptive statistics for key variables table. Variables \| Region \| \| \| Nature of property rights \|; \| Eastern region \| Central region \| Western region \| SOEs \| Private firms; $\text{Innovation}\text{ }$(mean) \| 121.772 \| 74.688 \| 67.597 \| 50.301 \| 19.095; $\text{Innovation}$ (median) \| 91 \| 46 \| 37 \| 45 \| 7; $\text{Fisdec}\text{ }$(mean) \| 0.727 \| 0.486 \| 0.392 \| 0.626 \| 0.659; $\text{Fisdec}$ (median) \| 0.626 \| 0.659 \| 0.626 \| 0.659 \| 0.626; Observed \| 6651 \| 1388 \| 1141 \| 2819 \| 6361 | |
| **Valuable comment 5**  With regard to models and inferences about the influence of local governments on SOEs vs. private companies, it would be reasonable to conduct separate models for these types of companies and also, in the initial models (before adding the mediation mechanism), include the interaction of ownership type with the level of decentralization | **Response to valuable comment 5**  I greatly appreciate your suggestion to conduct separate regression estimations for SOEs and private enterprises. The moderated mediation model used in this study can directly test the boundary conditions of the mediating mechanism. Rather than only showing that the effect of fiscal decentralization differs between SOEs and private enterprises, it explicitly examines “why and how ownership matters”, through the heterogeneous response to local government innovation preferences.  The interaction term between local government innovation preference and ownership type (*Prefer×State*, coefficient=6.676^***^) is statistically significant, indicating that the mediating mechanism varies across ownership types. This provides deeper theoretical insights than separate subsample baseline models.  Second, the full-sample model in this study uses a total of 9180 observations, allowing for precise estimation of firm fixed effects and year fixed effects. Splitting the sample would reduce statistical power. The current model maximizes the use of available data while still identifying ownership heterogeneity effects.  Third, our theoretical framework emphasizes that SOEs and private enterprises face different incentives and constraints within the same institutional environment. The moderated mediation model allows the effect of the core channel (local government innovation preference) to vary with ownership type while controlling for the overall effect of fiscal decentralization. This is consistent with the theoretical framework: ownership type moderates the translation of government preferences into innovation outputs, rather than fundamentally altering the relationship between fiscal decentralization and innovation.  In summary, the current approach not only identifies whether heterogeneous effects exist, but also clarifies the channels and directions of such differences, which is crucial for policy design under China’s unique institutional context. |
| **Valuable comments from Reviewer #2:** | |
| **Valuable comment 1**  Thank you for addressing all my previous comments. I really appreciate your efforts. Therefore, I recommend accepting this paper in its current version. | **Response to valuable comment 1**  We would like to express our sincere gratitude for recognizing our revision efforts and recommending acceptance of our manuscript in its current version. We deeply appreciate the time and dedication you devoted to providing thorough and constructive feedback throughout the review process. Your insightful comments have been invaluable in helping us strengthen our work and improve the overall quality of this paper. Thank you for your scholarly guidance and professional support. |
| We greatly appreciate your effort in reviewing this paper and your treasured comments provided for our paper, all of which would indeed help us to learn how to write a top-tier academic paper and how to enhance our manuscript. Although we have to make efforts to revise this paper, we indeed learn a lot. We are really appreciated your valuable comments and suggestions. Thank you so much. | |
